# Supplementary material for: Increasing global precipitation whiplash due to anthropogenic greenhouse gas emissions
Source: Nat Commun. 2023 May 18;14:2796. doi: 10.1038/s41467-023-38510-9 (PMC10195789; doi:10.1038/s41467-023-38510-9)
Supplement: Supplementary file 1 — Supplementary Information [file 41467_2023_38510_MOESM1_ESM.pdf]

Supplementary information for

Increasing global precipitation whiplash due to anthropogenic greenhouse gas emissions

Xuezhi Tan, Xinxin Wu, Zeqin Huang, Jianyu Fu, Xuejin Tan, Simin Deng, Yaxin Liu,

Thian Yew Gan, Bingjun Liu

Supplementary Text 1

In a given radiative forcing scenario, the ensemble model uncertainty of CESM-LENS consists of internal climate variability only, while that of CMIP6 composed of a combination of internal climate variability and model formulation differences (i.e., structural uncertainty) with unclear relative importance[1,2]. The prerequisite of using CMIP6 ensemble for comparison with CESM-LENS to enhance the robustness of the results is that we need to determine that the spread of the CMIP6 ensemble is well representative of the internal variability generated by CESM-LENS. First, we use an approach similar to Kay, et al. [3] to determine how much of the spread in the CMIP6 projections can be explained by internal climate variability alone. We assume that the CESM-LENS spread is across the range of internal variability. We calculate the trend in the frequency of precipitation whiplash for each member of CESM-LENS and CMIP6 in the current period (1979-2019) and future period (2060-2099), respectively, and then quantify the spread of frequency trends with the standard deviation of the trends across members. The *f* test is then used to evaluate whether the model spread of CMIP6 is statistically different from that of CESM-LENS. Supplementary Fig. 23 indicates that for the frequency of precipitation whiplash in most regions, regardless of the period, the trend spread estimated with CESM-LENS, which is generated by internal climate variability only, is not statistically significantly different from the trend spread within CMIP6 (areas not dotted in the CMIP6 panel in Fig. 23), implying that the CMIP6 spread over the vast majority of regions can be explained by the internal climate variability estimated by

CESM-LENS. The spatial pattern of the standard deviation of the annual mean trends within the two periods for the dry-to-wet and wet-to-dry events is similar for both ensembles. The magnitude of spread in the CMIP6 ensemble is larger (colors in the CMIP6 maps is darker) compared to CESM-LENS, which is consistent with the concept that CMIP6 contains contributions from model variability and internal climate variability.

In addition, we calculate the ratio of the standard deviation of the trend between CESM-LENS and CMIP6 members derived above (CESM-LENS divided by CMIP6), to simply quantify the contribution of internal variability to the spread of the trend in the CMIP6 ensemble, by referring to Deser, et al. [1]. A ratio greater (smaller) than 0.5 means that the contribution of internal variability is greater (smaller) compared to model variability, and a ratio > 0.75 means that the spread in the CESM-LENS trend is not significantly different from that in the CMIP6 trend. The results (not shown) show that for both whiplash events, internal climate variability is more important than structural differences of models in the current (future) period in 98% (95%) of the regions. In 83% (82%) of the regions, there is no statistically significant difference between the model spread of these two large ensembles. Therefore, the above analysis supports that the spread of the CMIP6 ensemble is representative of the internal variability generated by CESM-LENS in our study. The CMIP6 subset we selected is suitable for use with CESM-LENS to enhance the robustness of the results.

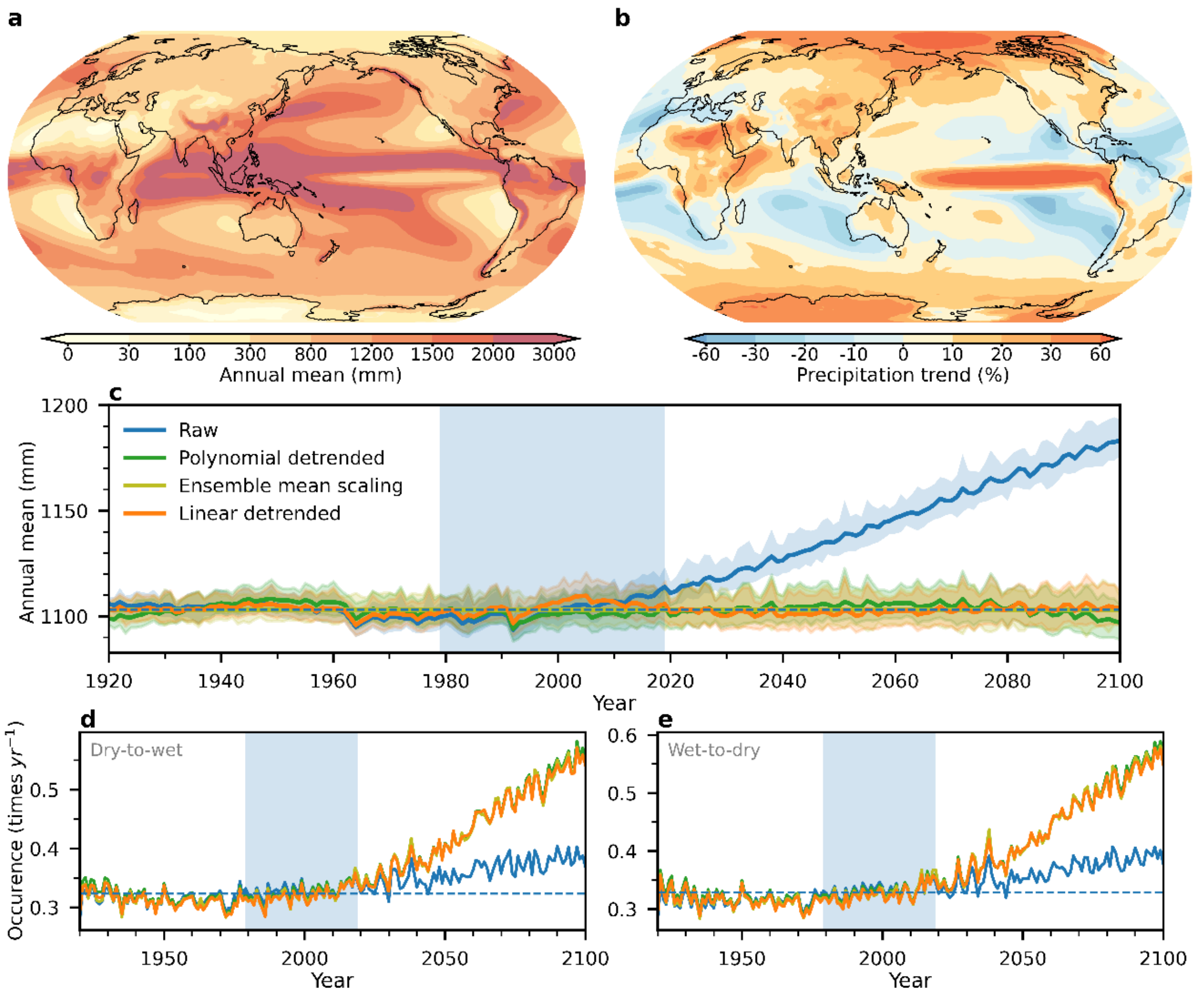

Supplementary Figure 1 Global (**a**) annual mean precipitation (mm) and (**b**) precipitation trend (%) over 1920-2100 in the CESM-LENS ensemble. (**c**), Time series of the original (blue), polynomial detrended (green), ensemble mean scaled (yellow) and linear detrended (orange) annual mean precipitation, shaded areas indicate the encompasses 90% of the full 40-member CESM-LENS ensemble spread. (**d-e**), the occurrence frequency of (**d**) dry-to-wet and (**e**) wet-to-dry whiplash calculated by the original (blue), polynomial detrended (green), ensemble mean scaled (yellow) and linear detrended (orange) precipitation.

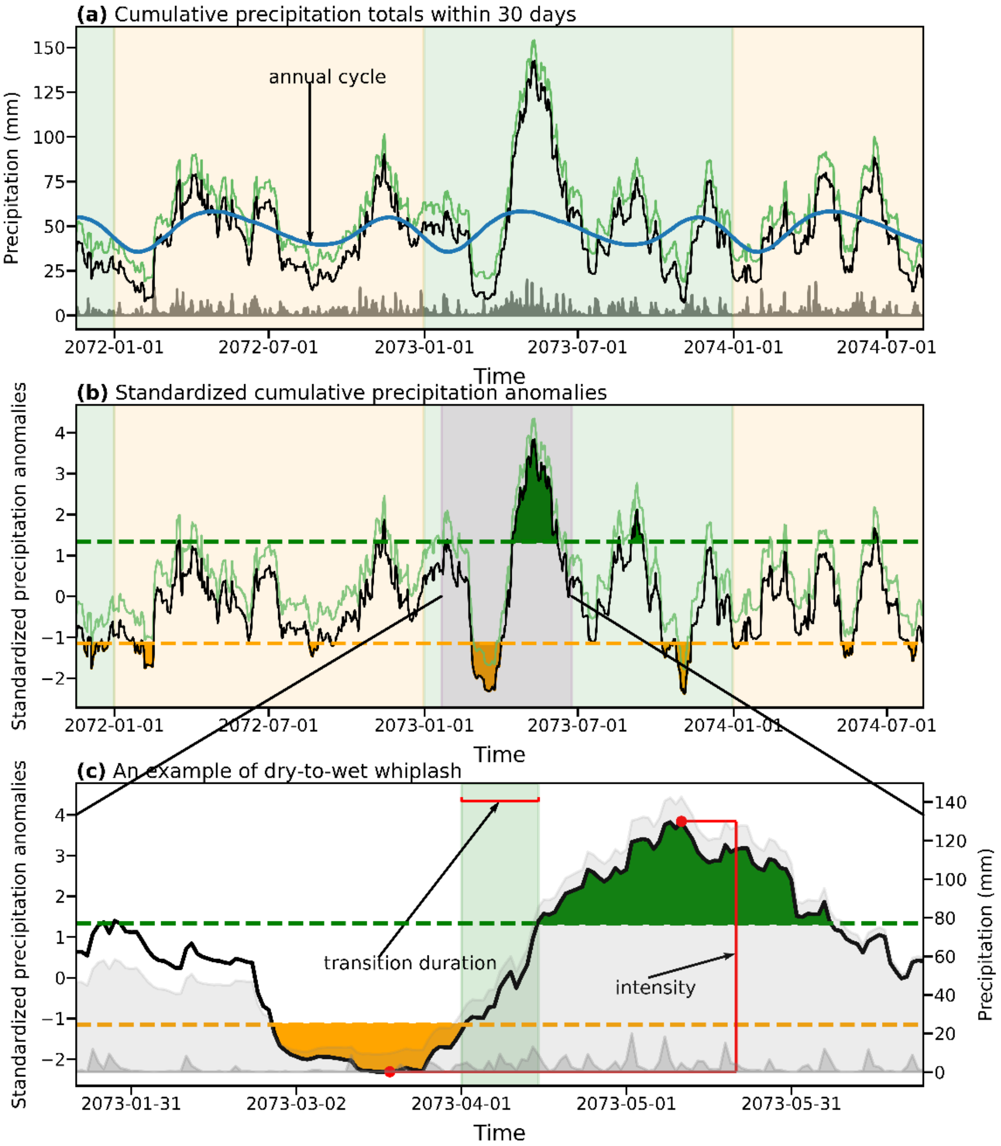

Supplementary Figure 2 An example illustrating the principle of the whiplash indices. **a**, the black (green) line indicates the detrended (raw) 30-day cumulative precipitation totals, the blue line indicates the annual cycle of cumulative precipitation totals, and the gray shading indicates the raw daily precipitation data. **b**, the standardized detrended (black) and raw (green) cumulative precipitation anomalies, and the yellow (green) dashed line indicates the 10th (90th) threshold over the current period (1979-2019; Methods). **c**, an example of dry-wet whiplash and the characteristics analyzed in this study. The light gray color indicates the detrended 30-day cumulative precipitation and the dark gray fill indicates the raw daily precipitation data.

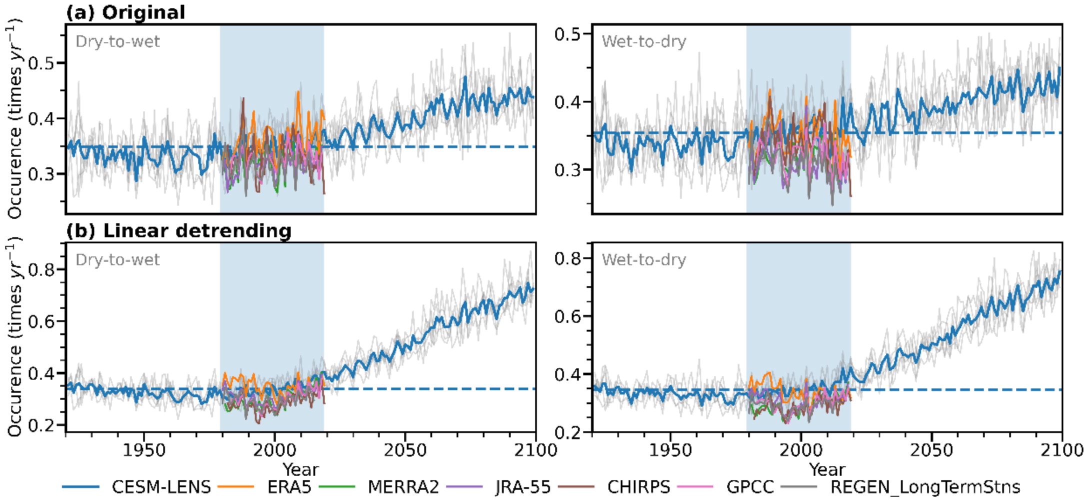

Supplementary Figure 3 The occurrence frequency of dry-to-wet (the left column) and (e) wet-to-dry (the right column) whiplash calculated by (a) original and (b) linear detrended precipitation in the 5 members of CESM-LENS ensemble and 6 gridded datasets.

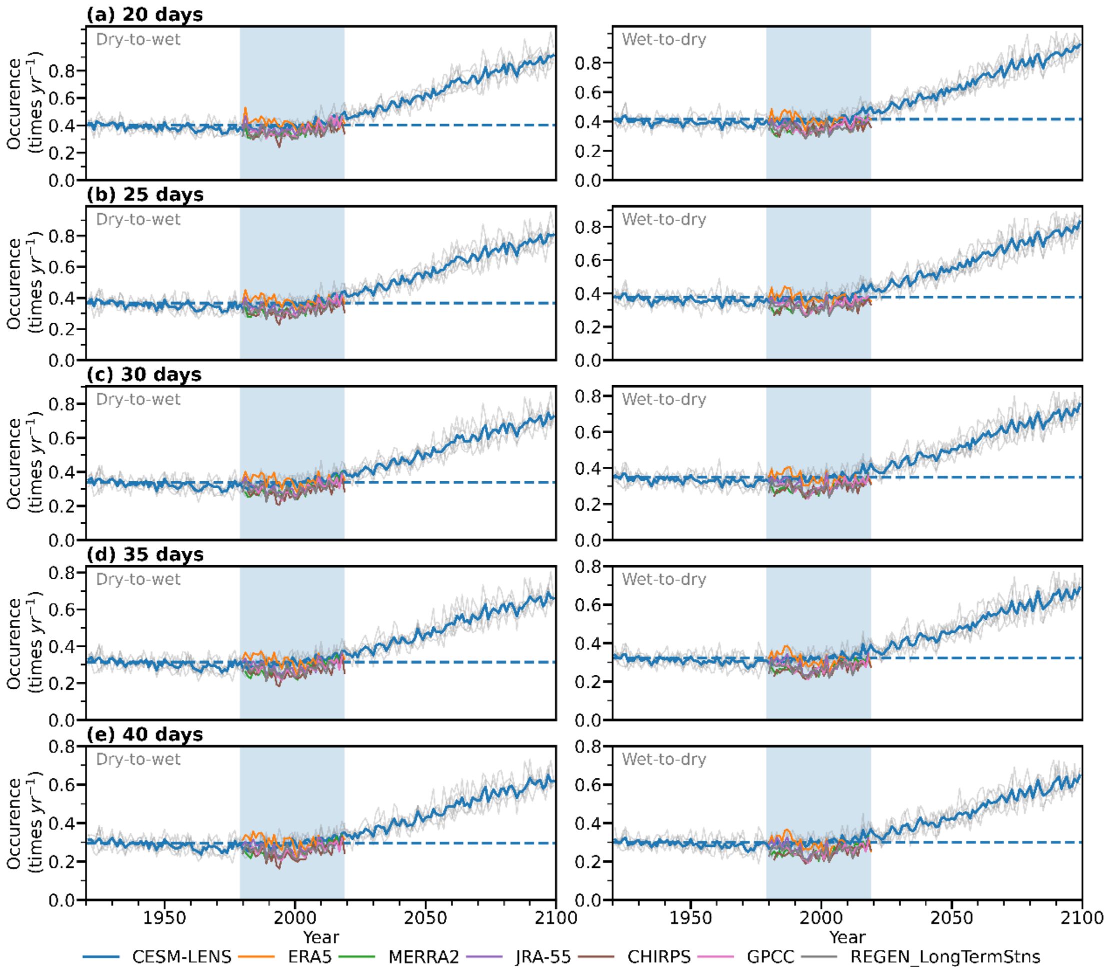

Supplementary Figure 4 The occurrence frequency of dry-to-wet (the left column) and (e) wet-to-dry (the right column) whiplash calculated by detrended cumulative precipitation within (**a**) 20, (**b**) 25, (**c**) 30, (**d**) 35, and (**e**) 40 days in the 5 members of CESM-LENS ensemble and 6 gridded datasets.

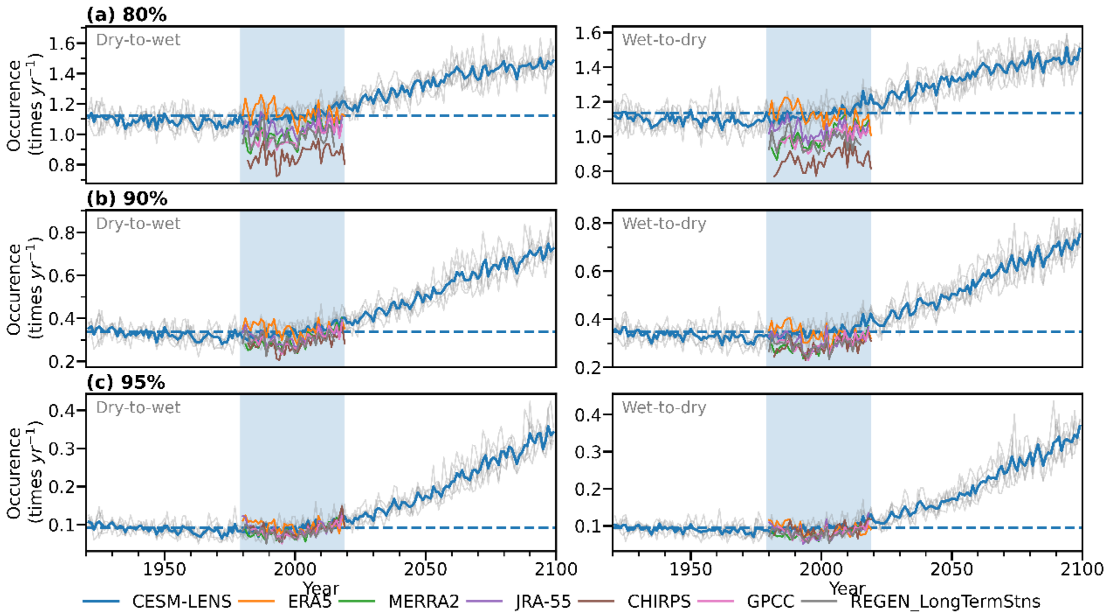

Supplementary Figure 5 The occurrence frequency of dry-to-wet (the left column) and (e) wet-to-dry (the right column) whiplash calculated with an extreme threshold of (**a**) 80th, (**b**) 90th, and (**c**) 95th quantile in the 5 members of CESM-LENS ensemble and 6 gridded datasets.

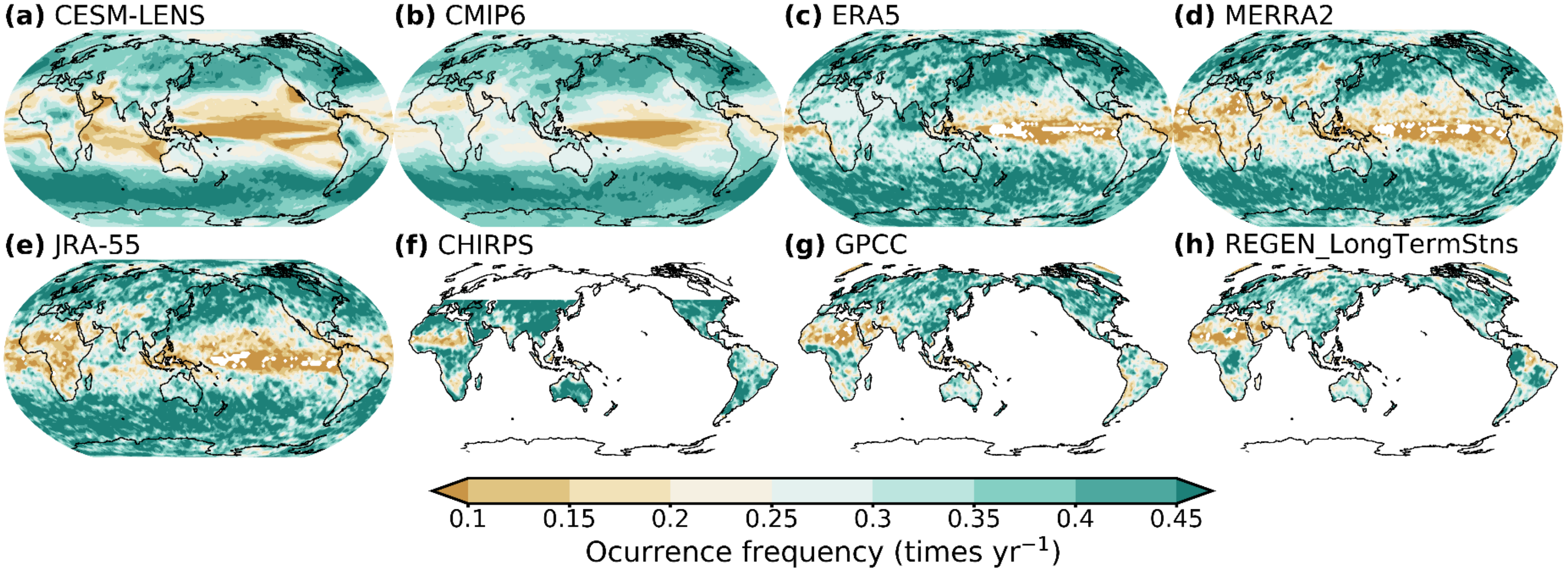

Supplementary Figure 6 Frequency of dry-to-wet whiplash in (**a**) CESM-LENS, (**b**) CMIP6, (**c-h**) 6 gridded datasets over 1979-2019.

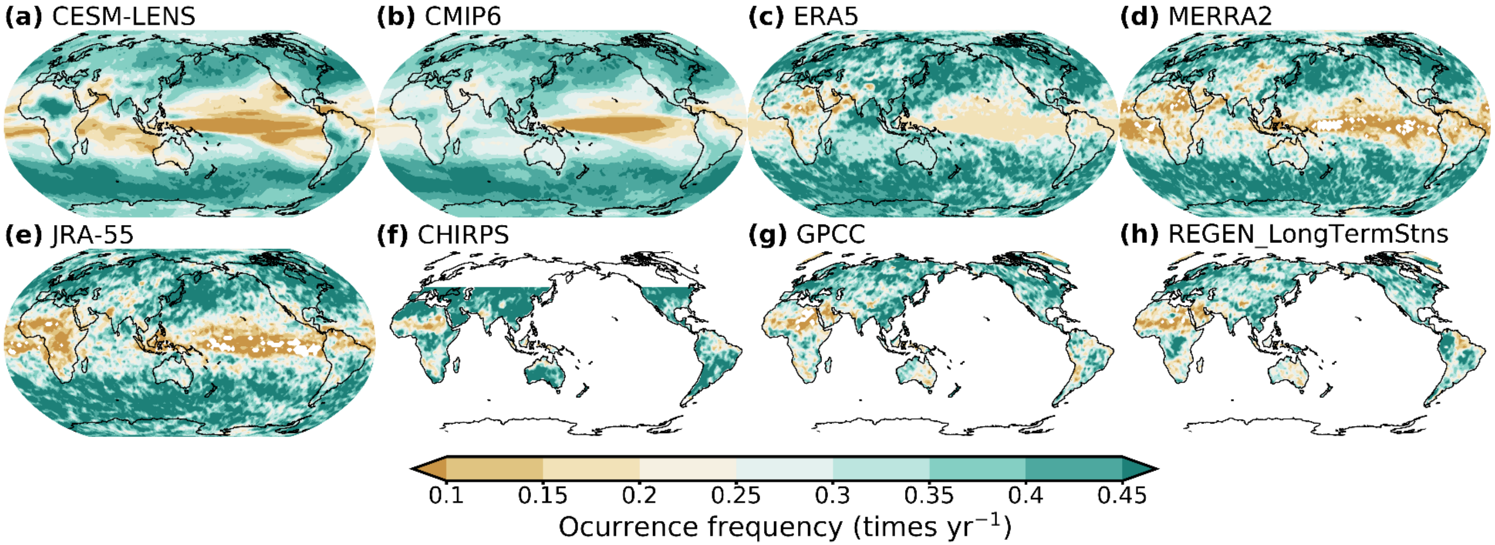

Supplementary Figure 7 Frequency of wet-to-dry whiplash in **(a)** CESM-LENS, **(b)** CMIP6, **(c-h)** 6 gridded datasets over 1979-2019.

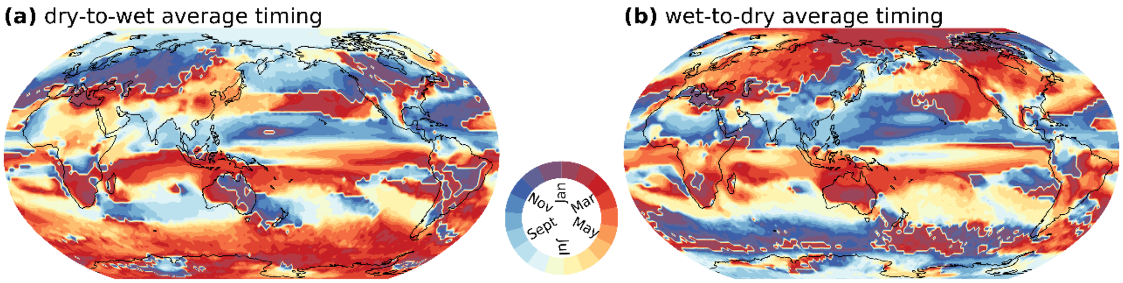

Supplementary Figure 8 Climatology of average timing of precipitation whiplash of (**a**) dry-to-wet and (**b**) wet-to-dry whiplash over the future period (2060-2099) in the CESM-LENS ensemble.

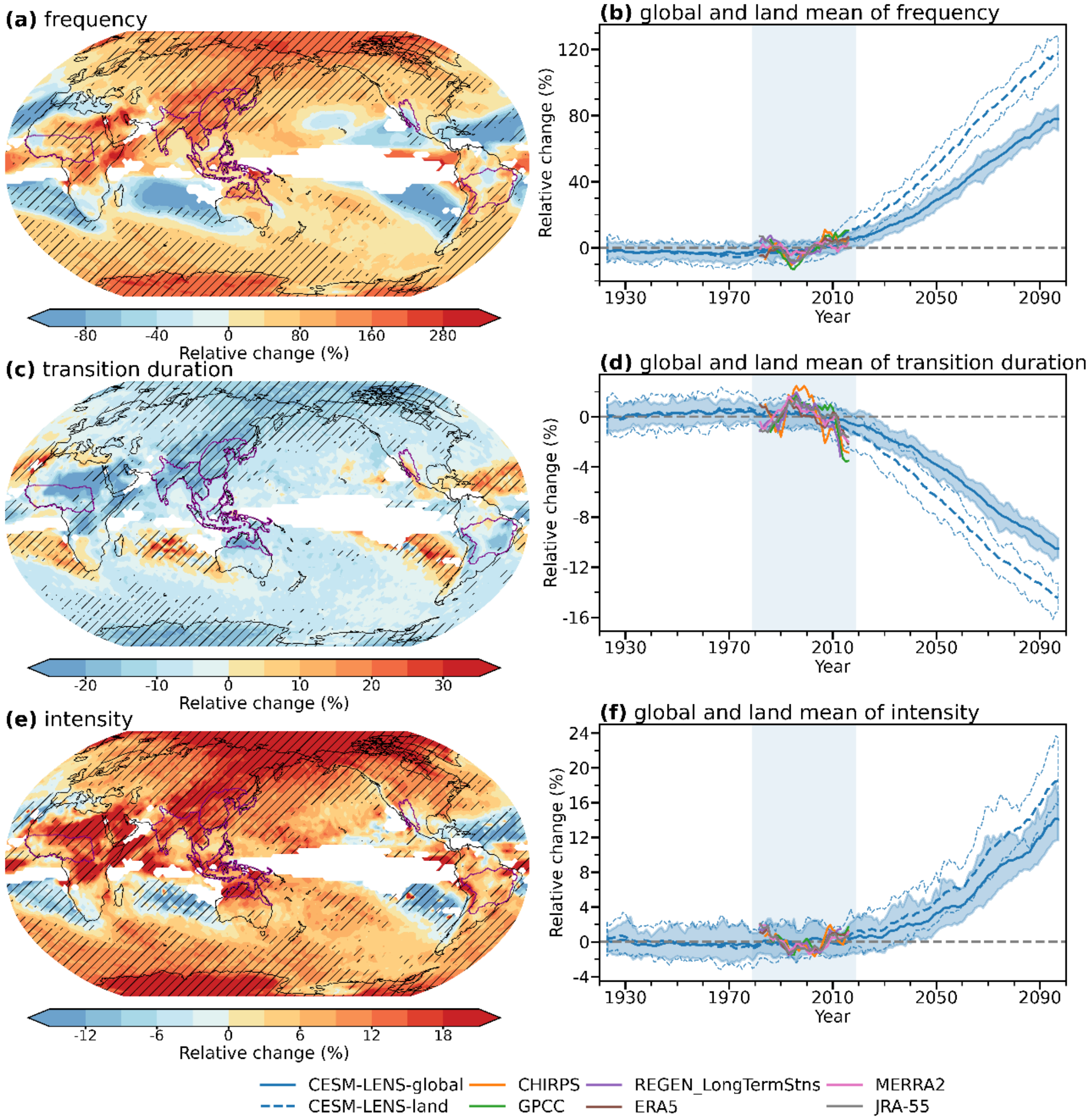

Supplementary Figure 9 Projected relative changes in the occurrence characteristics of **wet-to-dry whiplash** over 1921-2099. **a, c** and **e** show projected relative change (%) in (**a**) occurrence frequency, (**c**) transition duration and (**e**) intensity of wet-to-dry whiplash at the last four decades of the 21st Century (2060-2099) under the RCP8.5 forcing relative to the current period (1979-2019). Hatching area shows that more than 90% of the members agree on the changes in occurrence characteristics across the 40-member **CESM-LENS ensemble**. Regions with fewer than five whiplash events in total for the 40 ensemble members in the current period are masked. CESM-LENS ensemble mean of global area-weighted average relative changes (%) in (**b**)

occurrence frequency, (**d**) transition duration and (**f**) intensity of wet-to-dry whiplash (dashed line for land mean and solid line for global mean). Time series are moving averaged over 5-year intervals. The shaded area shows the spread of 90% of the ensemble. The baseline (grey dashed horizontal line) for the relative changes in the occurrence characteristics of whiplash is respective mean value over the current period (1979-2019). Note that CHIRPS has a coverage of 50° S-50° N land area, GPCC and REGEN_LongTermStns have a coverage of land only.

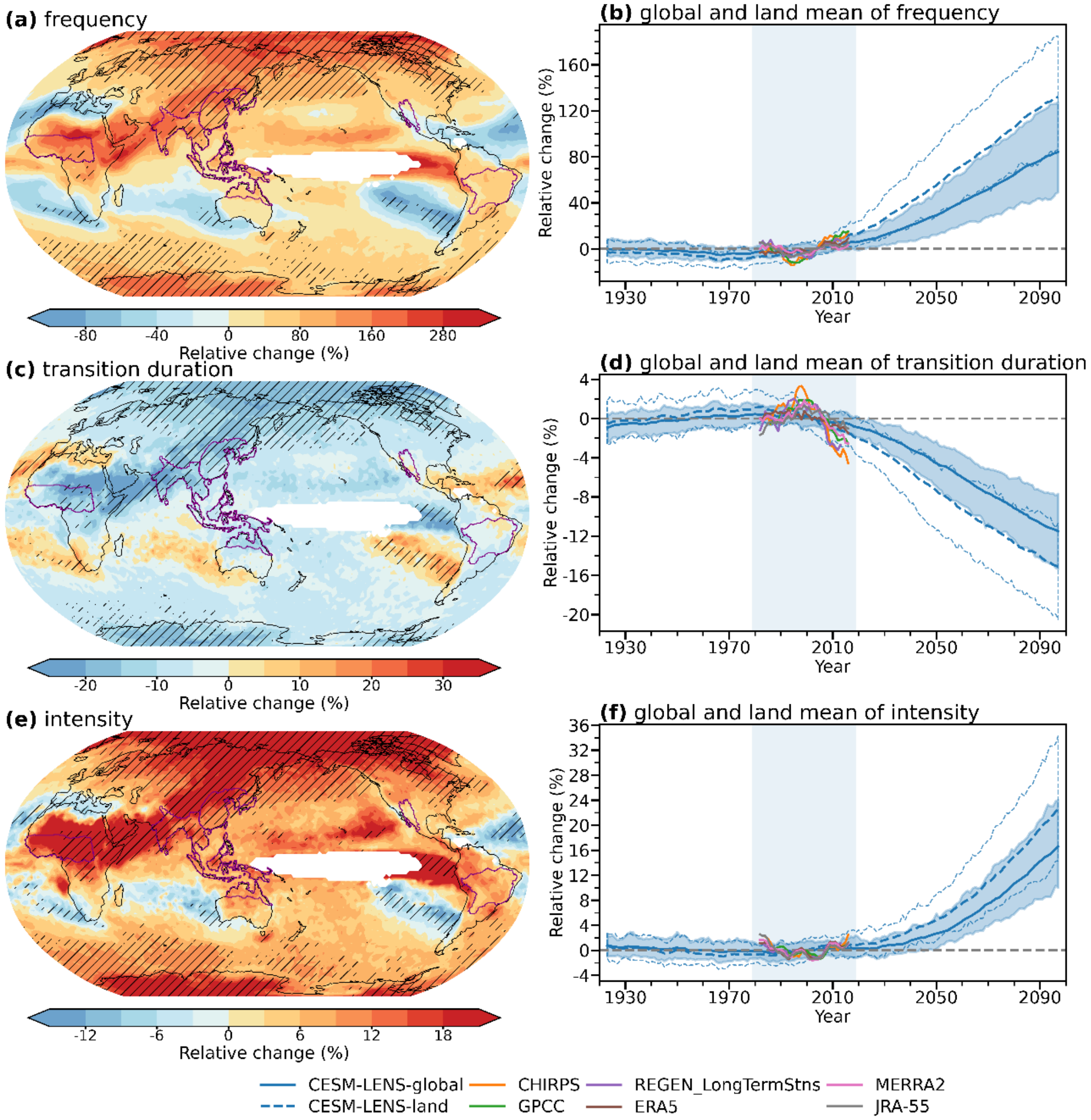

Supplementary Figure 10 Projected relative changes in the occurrence characteristics of **dry-to-wet whiplash** over 1921-2099. **a, c** and **e** show projected relative change (%) in (**a**) occurrence frequency, (**c**) transition duration and (**e**) intensity of dry-to-wet whiplash at the last four decades of the 21st Century (2060-2099) under the SSP5-8.5 forcing relative to the current period (1979-2019). Hatching shows more than 90% of the members agree on the changes in occurrence characteristics across the 55-member **CMIP6 ensemble**. Regions with fewer than five whiplash events in total for the 55 ensemble members in the current period are masked. The CMIP6 ensemble mean value of global area-weighted average relative changes (%) in (**b**) occurrence

frequency, (**d**) transition duration and (**f**) intensity of dry-to-wet whiplash (dashed line for land mean and solid line for global mean). Time series are moving averaged over 5-year intervals. The shaded area shows the spread of 90% of the ensemble. The baseline (grey dashed horizontal line) for the relative changes in the occurrence characteristics of whiplash is respective mean value over the current period (1979-2019). Note that CHIRPS has a coverage of 50° S-50° N land area, GPCC and REGEN_LongTermStns have a coverage of land only.

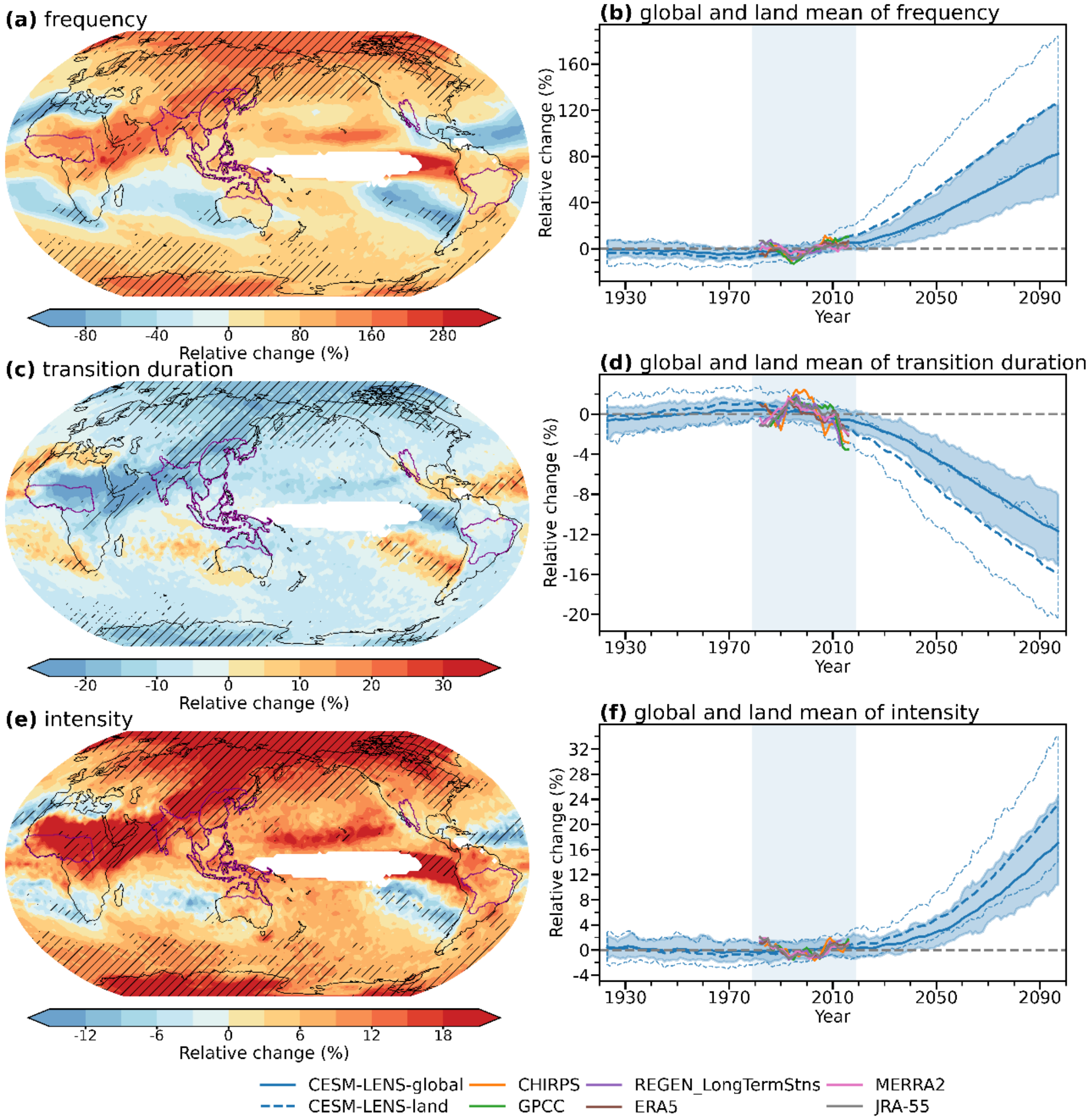

Supplementary Figure 11 Projected relative changes in the occurrence characteristics of **wet-to-dry whiplash** over 1921-2099. **a, c** and **e** show projected relative change (%) in (**a**) occurrence frequency, (**c**) transition duration and (**e**) intensity of wet-to-dry whiplash at the last four decades of the 21st Century (2060-2099) under the SSP5-8.5 forcing relative to the current period (1979-2019). Hatching shows more than 90% of the members agree on the changes in occurrence characteristics across the 55-member **CMIP6 ensemble**. Regions with fewer than five whiplash events in total for the 55 ensemble members in the current period are masked. The CMIP6 ensemble mean value of global area-weighted average relative changes (%) in (**b**) occurrence

frequency, (**d**) transition duration and (**f**) intensity of wet-to-dry whiplash (dashed line for land mean and solid line for global mean). Time series are moving averaged over 5-year intervals. The shaded area shows the spread of 90% of the ensemble. The baseline (grey dashed horizontal line) for the relative changes in the occurrence characteristics of whiplash is respective mean value over the current period (1979-2019). Note that CHIRPS has a coverage of 50° S-50° N land area, GPCC and REGEN_LongTermStns have a coverage of land only.

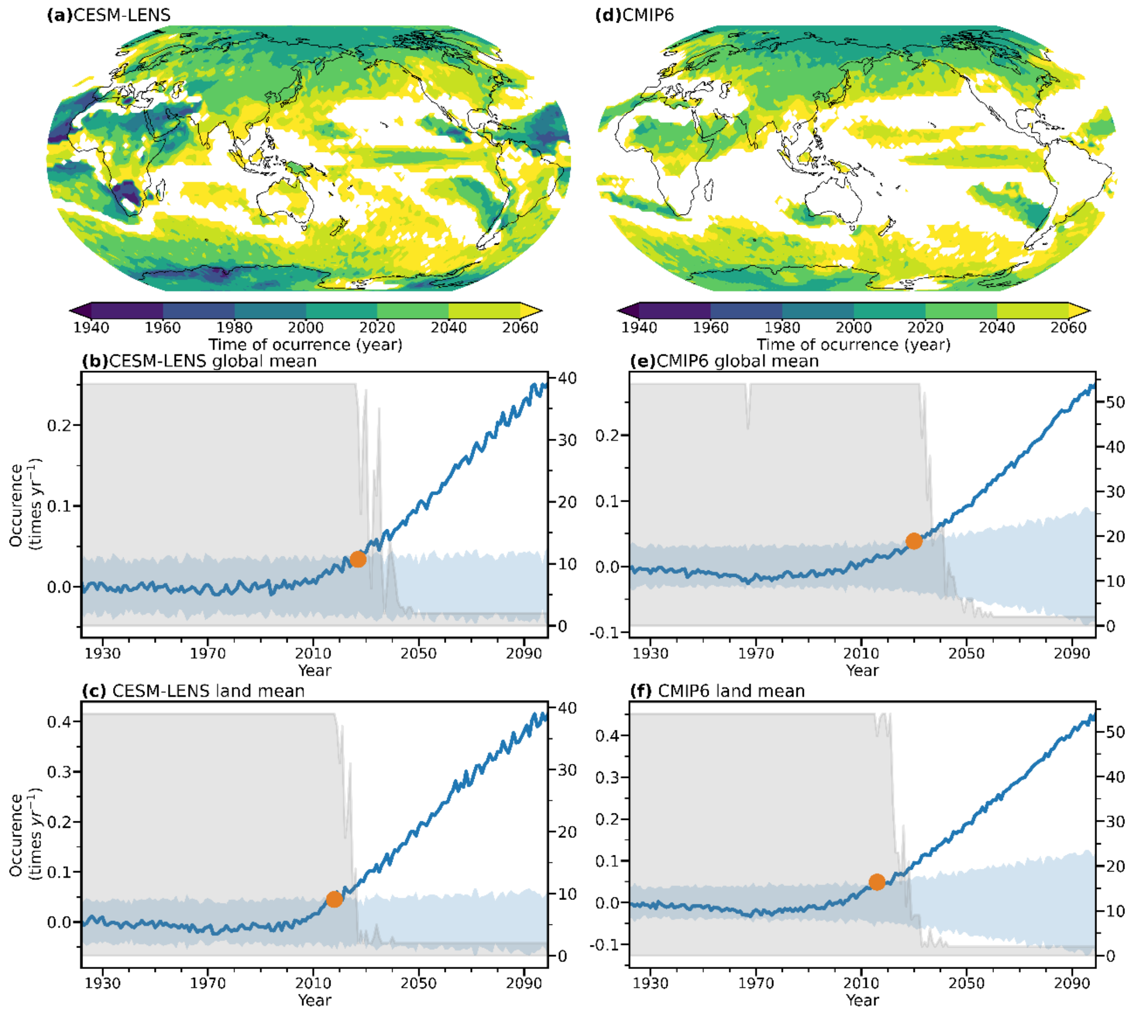

Supplementary Figure 12 Maps showing the first year when the forced response of **dry-to-wet whiplash** is greater than the uncertainties, i.e., the S/N is greater or equal to 1, in (**a**) CESM-LENS and (**d**) CMIP6 ensembles. The 10-year running mean (**b** and **e**) global and (**c** and **f**) land mean forced response (blue line) and the uncertainties standard deviation (blue shading) in CESM-LENS (left column) and CMIP6 (right column) are represented. We proceed by random resampling (Methods) to define the number of members required to have 95% of chance to produce a robust whiplash change (S/N ≥ 1) (the number ranges thus from 2-40 for CESM-LENS and from 2-54 for CMIP6; grey shading). Orange circles are the first year when the forced response is greater than the uncertainties, i.e., the absolute value of S/N is greater or equal to 1.

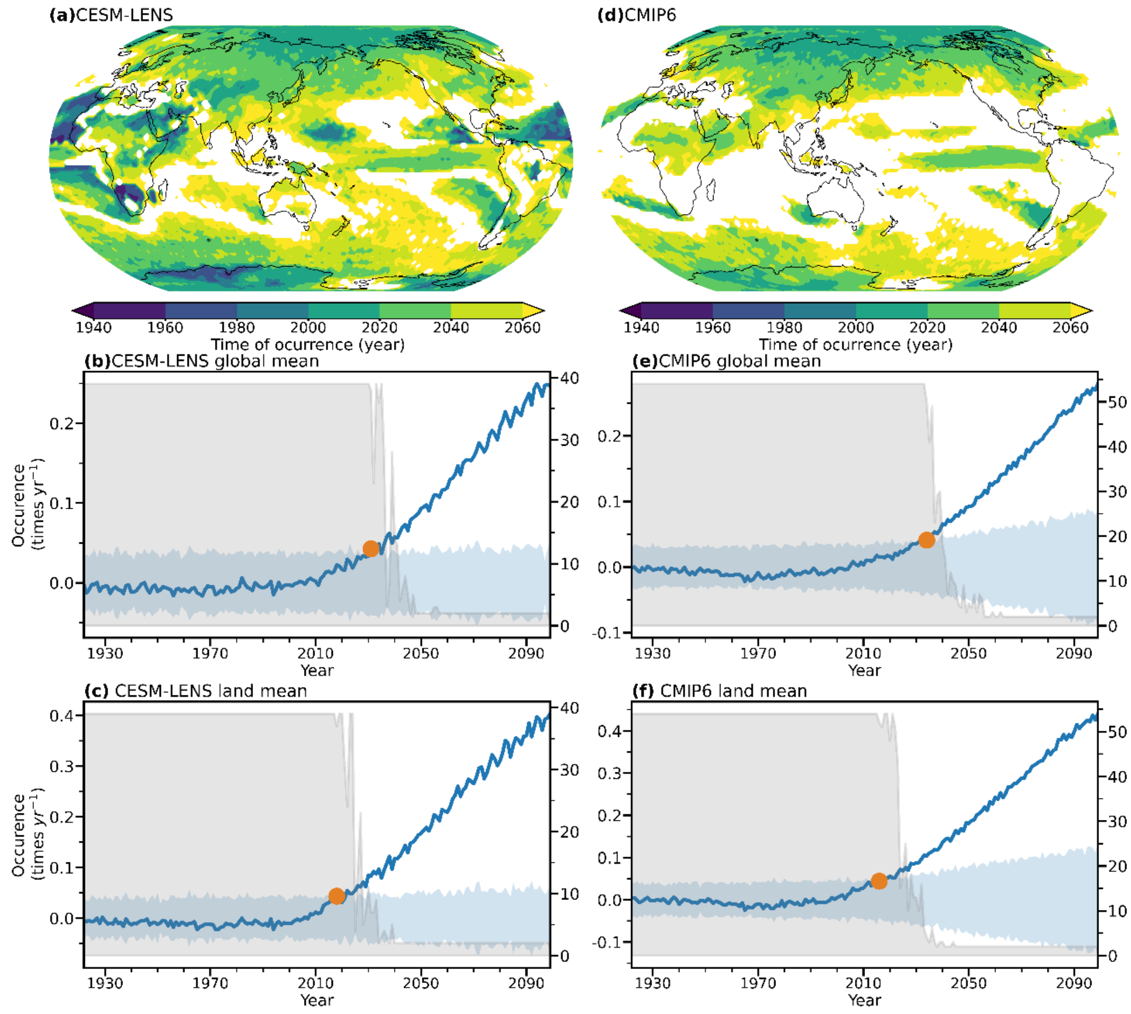

Supplementary Figure 13 Maps showing the first year when the forced response of **wet-to-dry whiplash** is greater than the uncertainties, i.e., the S/N is greater or equal to 1, in (**a**) CESM-LENS and (**d**) CMIP6 ensembles. The 10-year running mean (**b** and **e**) global and (**c** and **f**) land mean forced response (blue line) and the uncertainties standard deviation (blue shading) in CESM-LENS (left column) and CMIP6 (right column) are represented. We proceed by random resampling (Methods) to define the number of members required to have 95% of chance to produce a robust whiplash change (S/N $\geq$ 1) (the number ranges thus from 2-40 for CESM-LENS and from 2-54 for CMIP6; grey shading). Orange circles are the first year when the forced response is greater than the uncertainties, i.e., the absolute value of S/N is greater or equal to 1.

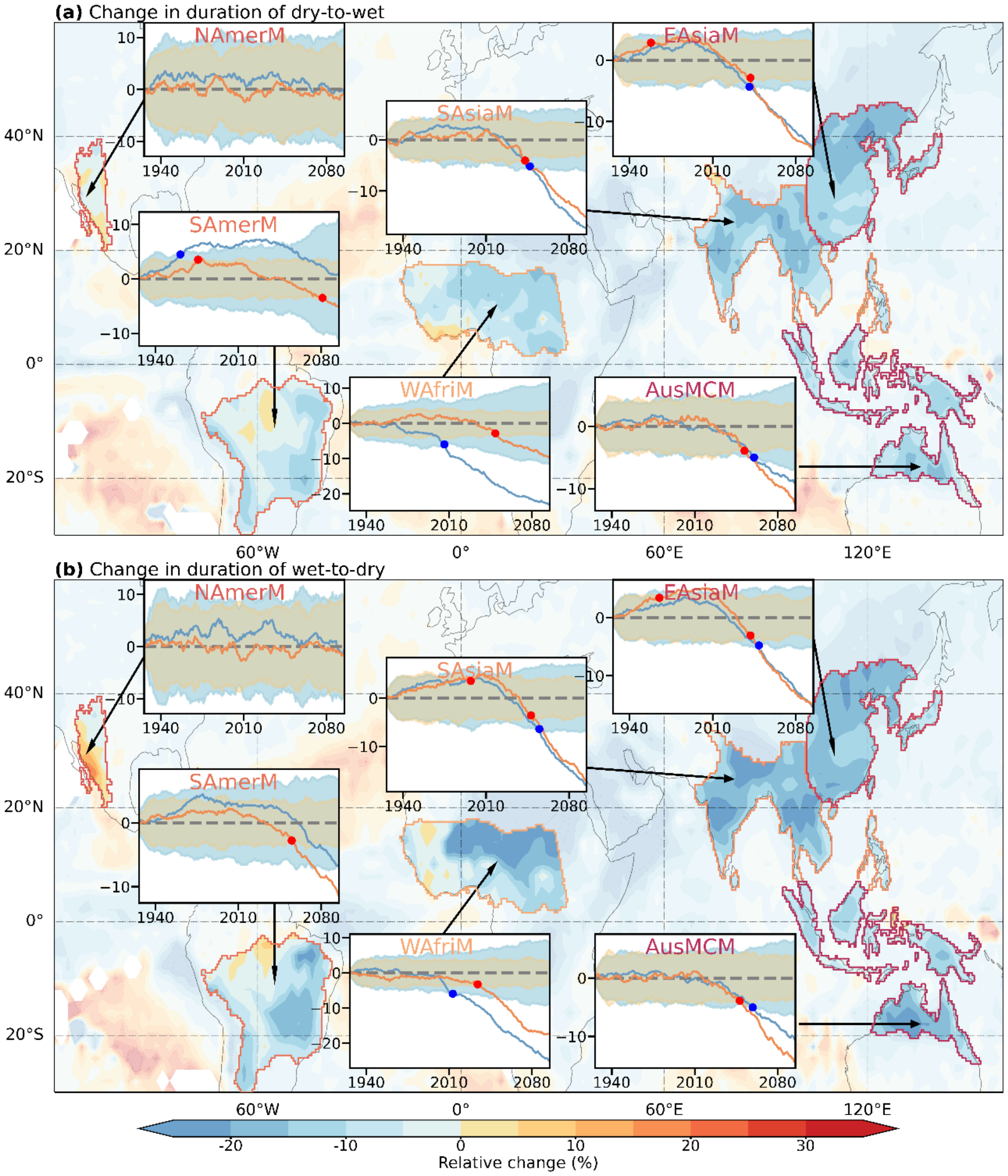

Supplementary Figure 14 Projected relative changes in the regional **transition duration** of precipitation whiplash. Relative changes (%) in transition duration of dry-to-wet whiplash (**a**), and wet-to-dry whiplash (**b**) events at the last four decades of the 21st Century (2060–2100) under the RCP8.5 forcing relative to the recent four decades (1979–2019) over six monsoon regions. The six monsoon regions are North American monsoon (NAmerM), South American monsoon (SAmerM),

West African monsoon (WAfriM), South and Southeast Asian monsoon (SAsiaM), East Asian monsoon (EAsiaM) and Australian-Maritime Continent monsoon (AusMCM) regions. Plots on maps indicate the area-weighted average changes in transition duration of precipitation whiplash in all monsoon regions derived from the ensemble mean of CMIP6 and CESM-LENS. Data are smoothed over 10-year intervals. The regional mean forced response (lines) and the uncertainties standard deviation (shadings) in CESM-LENS (orange) and CMIP6 (blue) are represented. Orange (blue) circles are the first year when the forced response is greater than the uncertainties, i.e., the absolute value of S/N is greater than or equal to 1, in CESM-LENS (CMIP6).

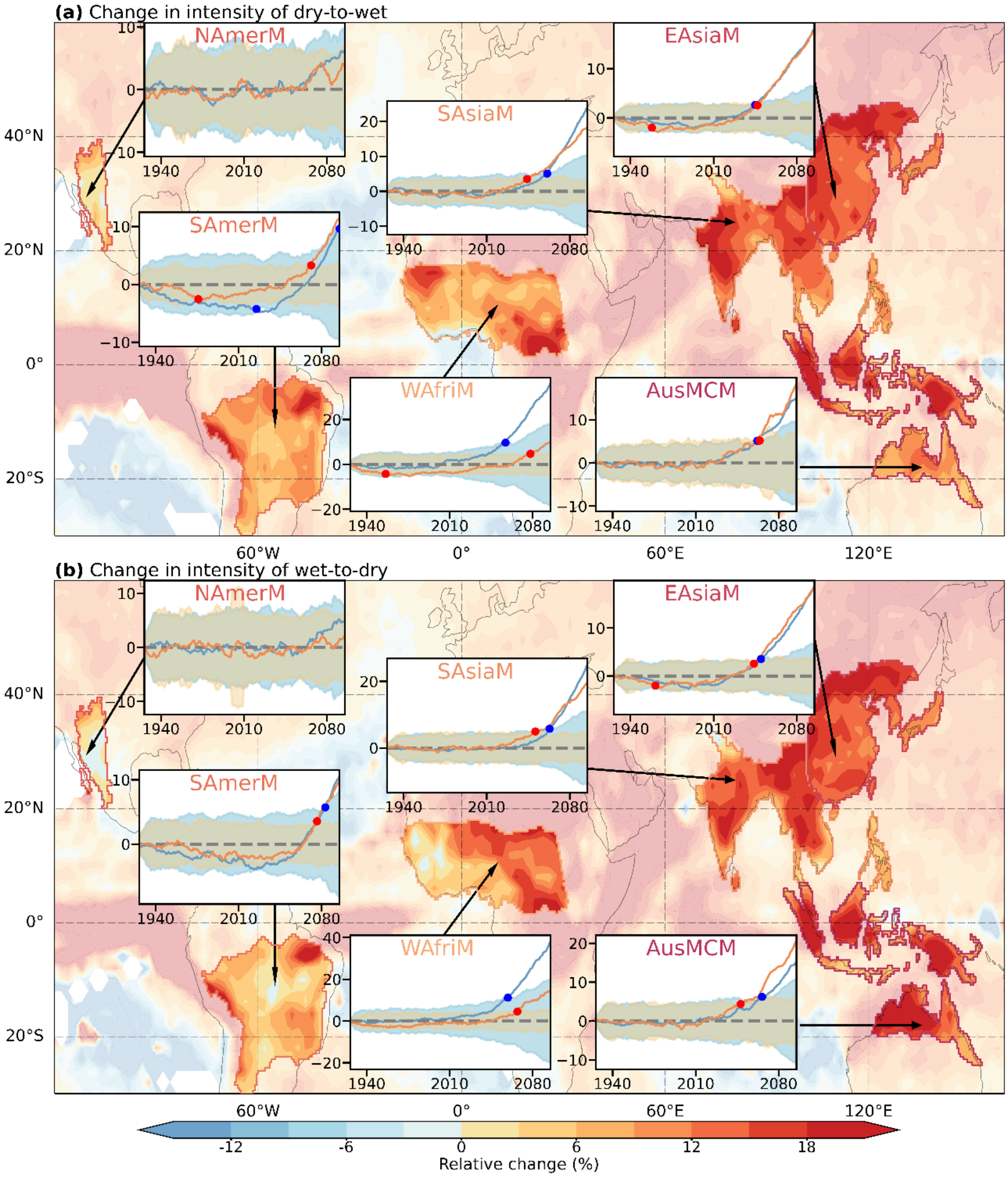

Supplementary Figure 15 Projected relative changes in the regional **occurrence intensity** of precipitation whiplash. Relative changes (%) in occurrence intensity of dry-to-wet whiplash (**a**), and wet-to-dry whiplash (**b**) events at the last four decades of the 21st Century (2060–2100) under the RCP8.5 forcing relative to the recent four decades (1979–2019) over six monsoon regions. The six monsoon regions are North American monsoon (NAmerM), South American monsoon

(SAmerM), West African monsoon (WAfriM), South and Southeast Asian monsoon (SAsiaM), East Asian monsoon (EAsiaM) and Australian-Maritime Continent monsoon (AusMCM) regions. Plots on maps indicate the area-weighted average changes in occurrence intensity of precipitation whiplash in all monsoon regions derived from the ensemble mean of CMIP6 and CESM-LENS. Data are smoothed over 10-year intervals. The regional mean forced response (lines) and the uncertainties standard deviation (shadings) in CESM-LENS (orange) and CMIP6 (blue) are represented. Orange (blue) circles are the first year when the forced response is greater than the uncertainties, i.e., the absolute value of S/N is greater than or equal to 1, in CESM-LENS (CMIP6).

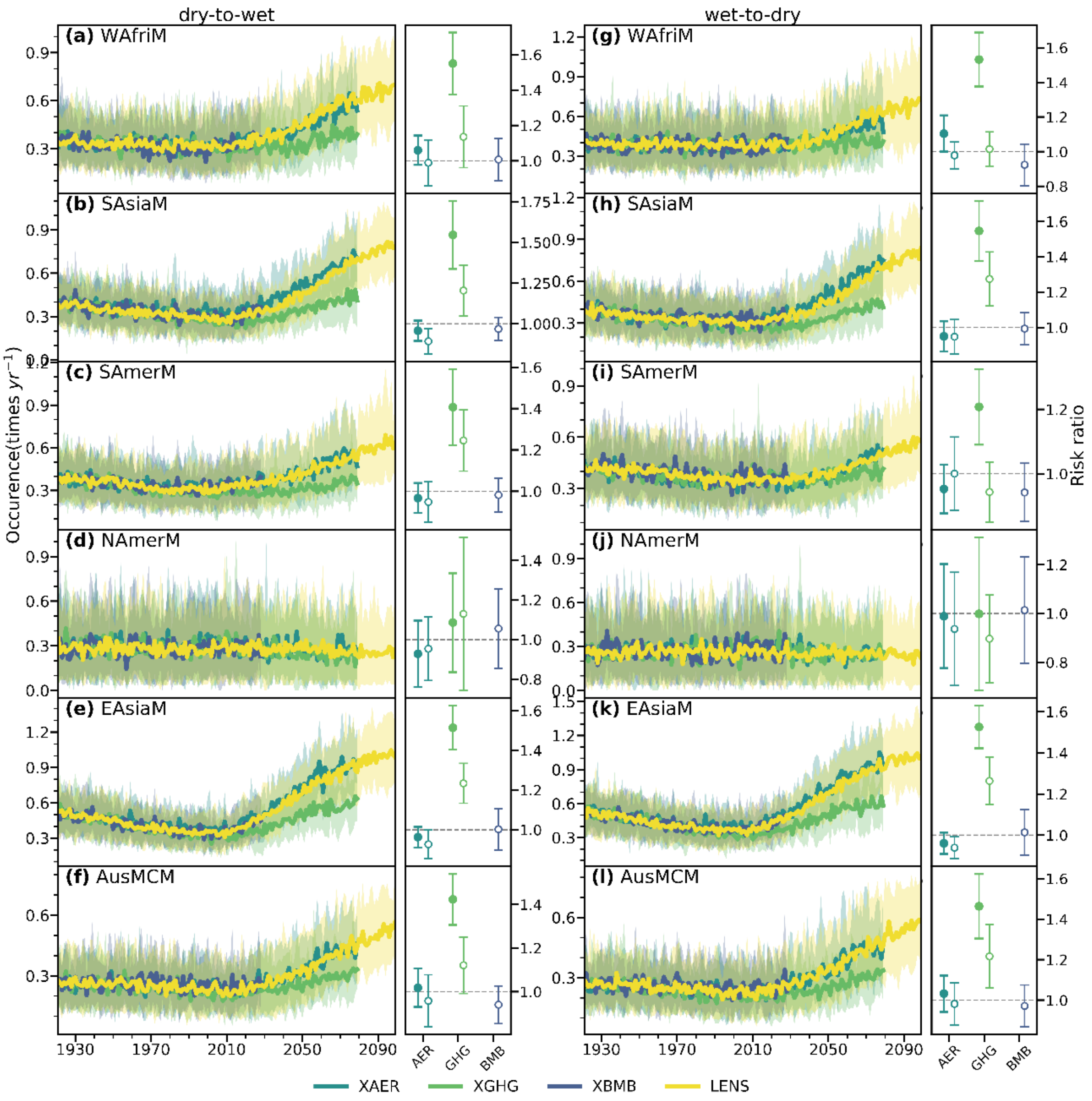

Supplementary Figure 16 Anthropogenic effects of changes in **occurrence frequency** of precipitation whiplash in monsoon regions. Time series of the regional area-weighted average of the occurrence frequency of dry-to-wet whiplash (**a**) and wet-to-dry whiplash (**b**) derived from the ensembles of CESM-LENS (yellow), all-but-no industrial aerosols (XAERs, cyan), all-but-no greenhouse gases (XGHGs, green), and all-but-no biomass burning aerosols (XBMB, purple). Shading area shows the spread of results derived from the 90% of the ensemble members. The circles at the right of each plot in **a** and **d** indicate the global average risk ratio of different

anthropogenic forcings on the global occurrence frequency of precipitation whiplash shown in the CESM-LENS by 2028 (hollow circles) and 2079 (solid circles), and the length of the stick indicates the inter-member standard deviation.

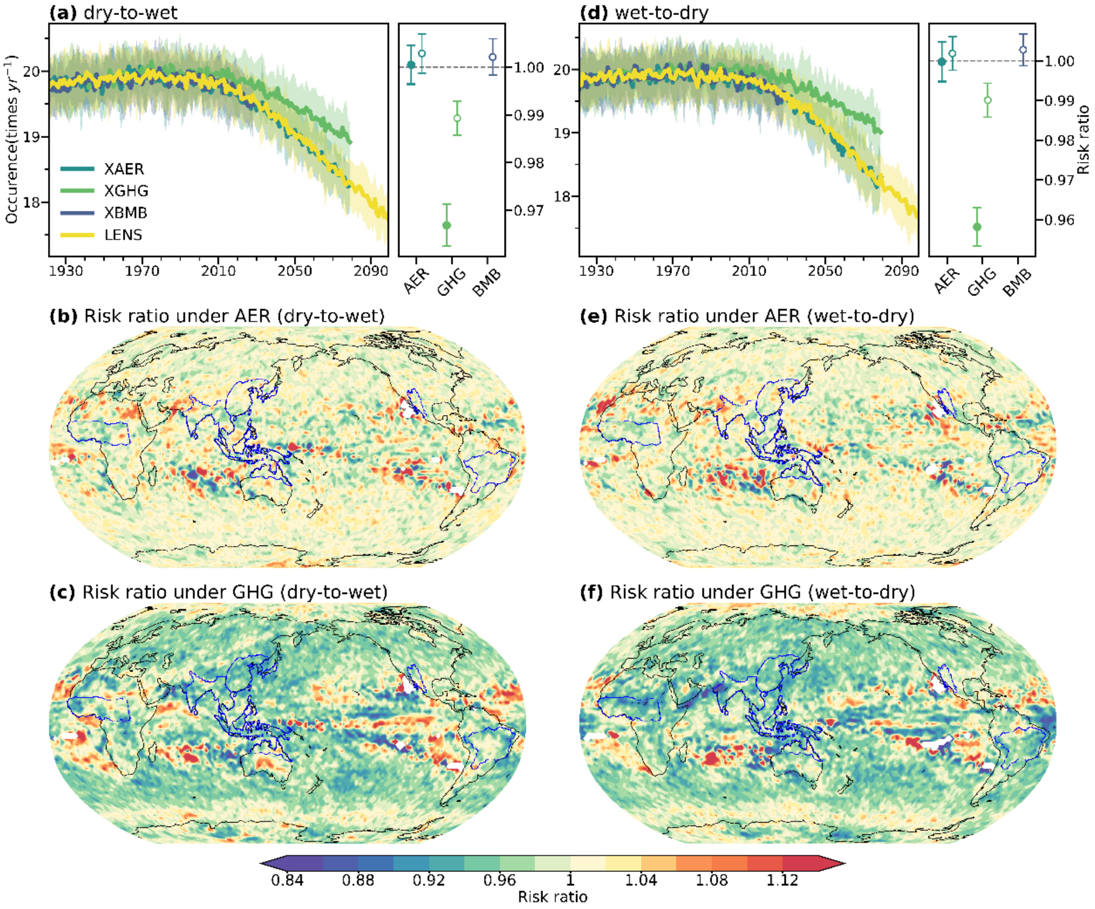

Supplementary Figure 17 Anthropogenic effects on changes in the **transition duration** of precipitation whiplash. Time series of the global area-weighted average of transition duration of dry-to-wet whiplash (**a**) and wet-to-dry whiplash (**d**) derived from the ensembles of CESM-LENS (yellow), all-but-no industrial aerosols (XAERs, cyan), all-but-no greenhouse gases (XGHGs, green), and all-but-no biomass burning aerosols (XBMB, purple). Shading area shows the spread of results derived from 90% of the ensemble members. The circles at the right of each plot in **a** and **d** indicate the global average risk ratio of different anthropogenic forcings on the global transition duration of precipitation whiplash shown in the CESM-LENS by 2028 (hollow circles) and 2079 (solid circles), and the length of the stick indicates the inter-member standard deviation. Maps are the mean risk ratio under AER (**b** and **e)** and GHG (**c** and **f)** on changes in the transition duration of dry-to-wet whiplash (**b-c**) and wet-to-dry whiplash (**e-f**) over 2040–2079. Hatching shows that more than 90% of ensemble members agree on the sign of risk ratio.

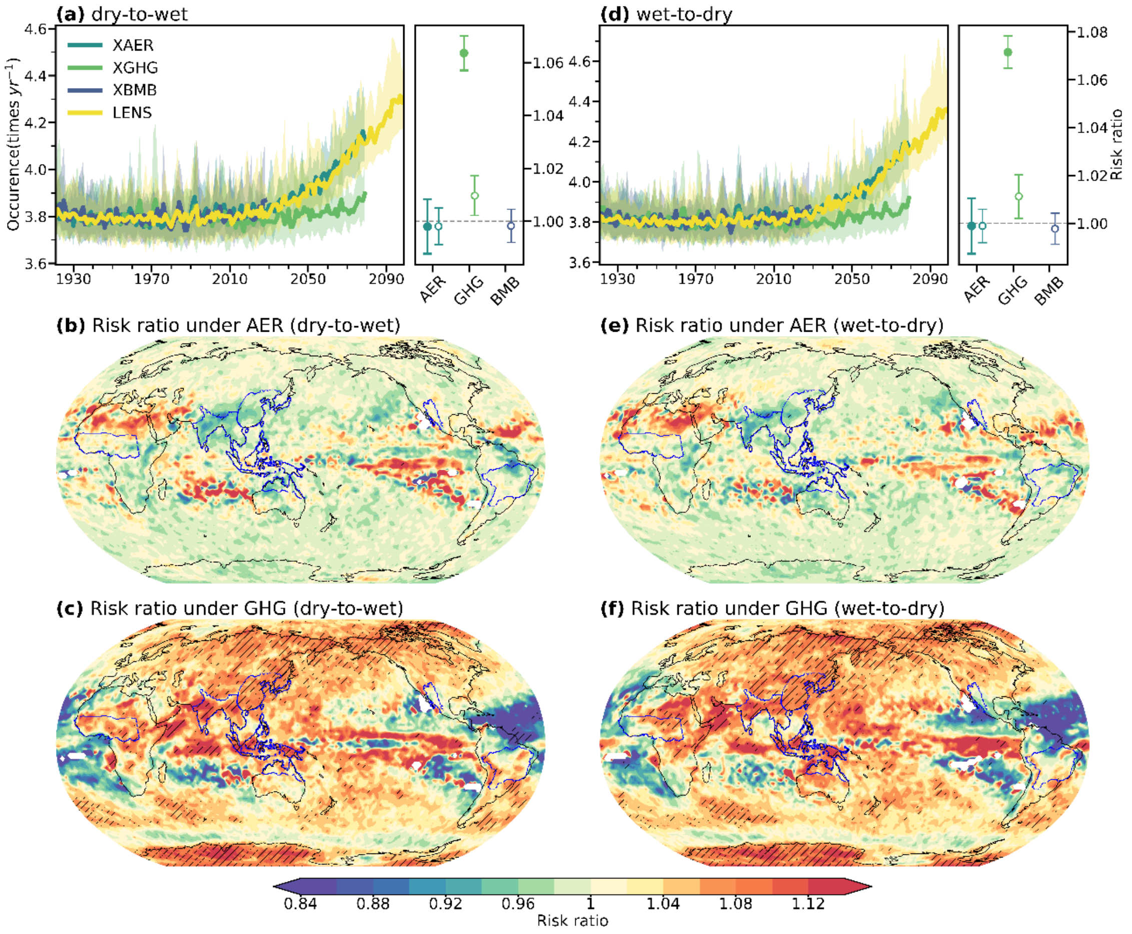

Supplementary Figure 18 Anthropogenic effects on changes in the **transition intensity** of precipitation whiplash. Time series of the global area-weighted average of transition intensity of dry-to-wet whiplash (**a**) and wet-to-dry whiplash (**d**) derived from the ensembles of CESM-LENS (yellow), all-but-no industrial aerosols (XAERs, cyan), all-but-no greenhouse gases (XGHGs, green), and all-but-no biomass burning aerosols (XBMB, purple). Shading area shows the spread of results derived from 90% of the ensemble members. The circles at the right of each plot in **a** and **d** indicate the global average risk ratio of different anthropogenic forcings on the global transition intensity of precipitation whiplash shown in the CESM-LENS by 2028 (hollow circles) and 2079 (solid circles), and the length of the stick indicates the inter-member standard deviation. Maps are the mean risk ratio under AER (**b** and **e)** and GHG (**c** and **f)** on changes in the transition intensity of dry-to-wet whiplash (**b-c**) and wet-to-dry whiplash (**e-f**) over 2040–2079. Hatching shows that more than 90% of ensemble members agree on the sign of risk ratio.

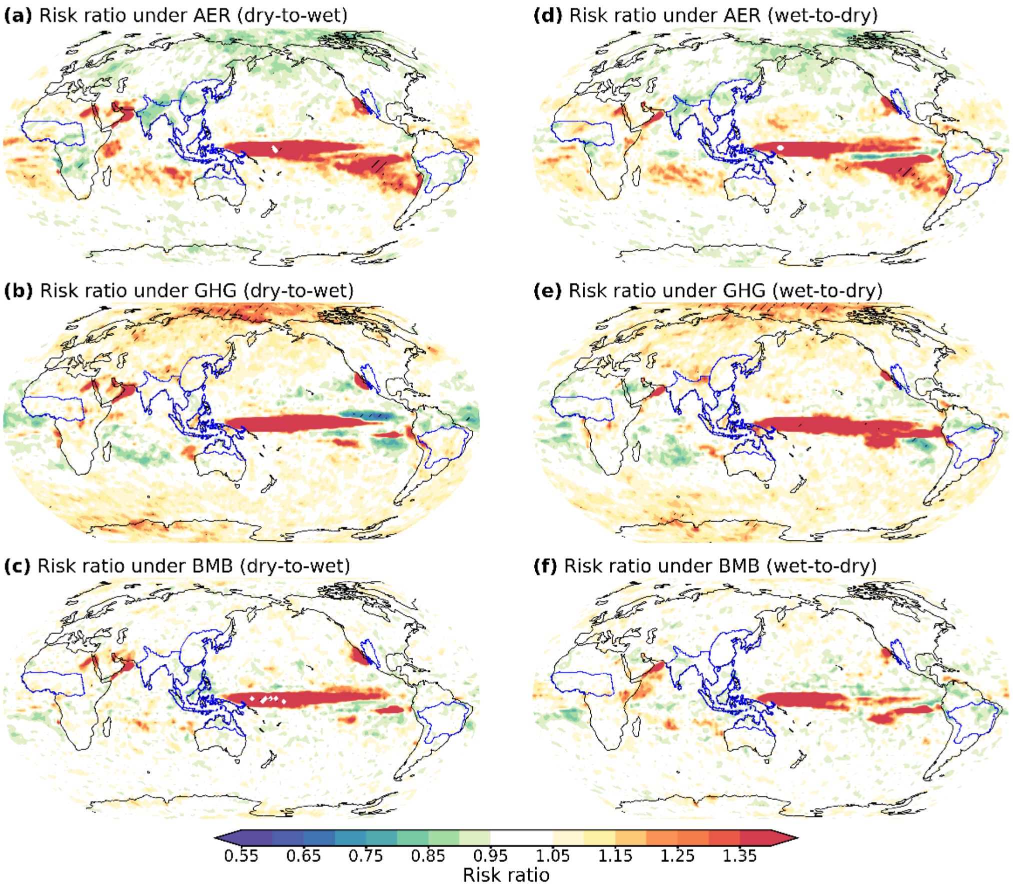

Supplementary Figure 19 Risk ratio of (**a, d)** AER, (**b, e)** GHG and (**c, f**) BMB on changes in occurrence frequency of dry-to-wet whiplash (left column) and wet-to-dry (right column) whiplash over 1921–2028. Hatching shows more than 90% of the ensemble members agree on the sign of relative influence.

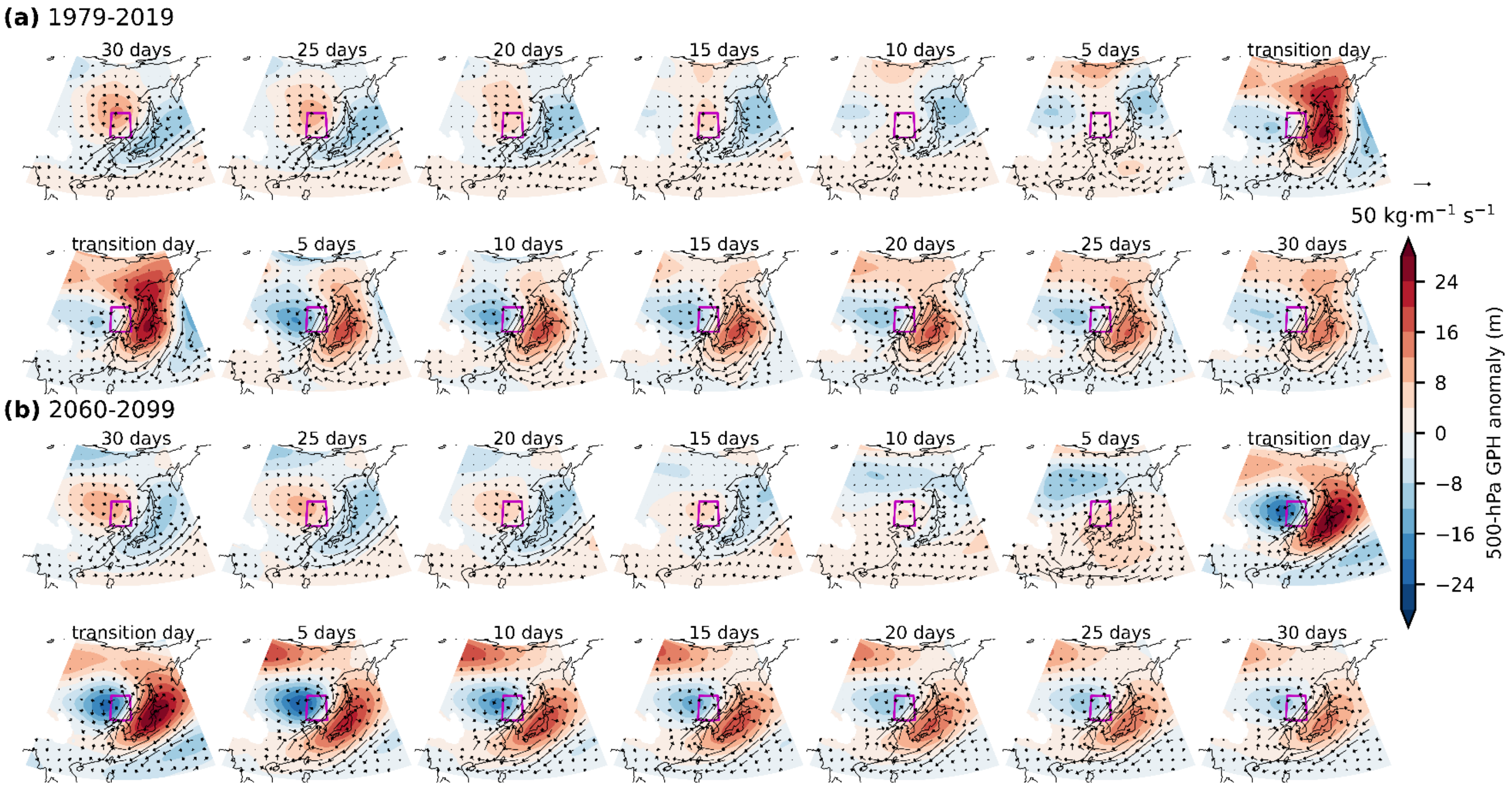

Supplementary Figure 20 Gradual changing process of large-scale atmospheric circulation of dry-to-wet whiplash over northeastern China (NEC). Average atmospheric anomalies for the 30, 25, 20, 15, 10 and 5 days before the occurrence of whiplash, the transition days, and the 5,10, 15, 20, 25, 30 days after the occurrence over the current period (1979-2019; **a**) and future period (2060-2099; **b**) in the CESM-LENS ensemble. Composite 500 hPa geopotential height (GPH) anomalies are shown by color and contour lines, and the magnitude and direction of the column-integrated water vapor anomalies are shown by scaled arrows

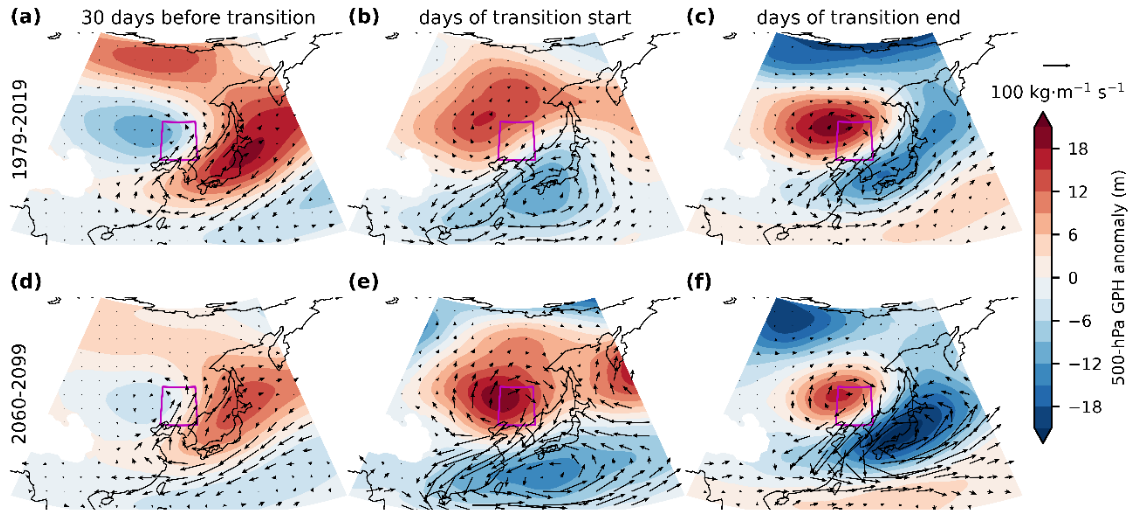

Supplementary Figure 21 Large scale atmospheric circulation associated with **wet-to-dry whiplash** over northeastern China. Average atmospheric anomalies for (**a** and **d**) 30 days before the occurrence of dry-to-wet whiplash (controlled by dry condition), (**b** and **e**) the days when the transition from dry to wet extremes starts, and (**c** and **f**) the days when the transition ends (the first days controlled by wet condition) over the current period (1979-2019; **a-c**) and the future period (2060-2099; **d-f**). Composite 500 hPa geopotential height (GPH) anomalies are shown by colorful shadings, and the magnitude and direction of the column-integrated water vapor anomalies are shown by scaled arrows.

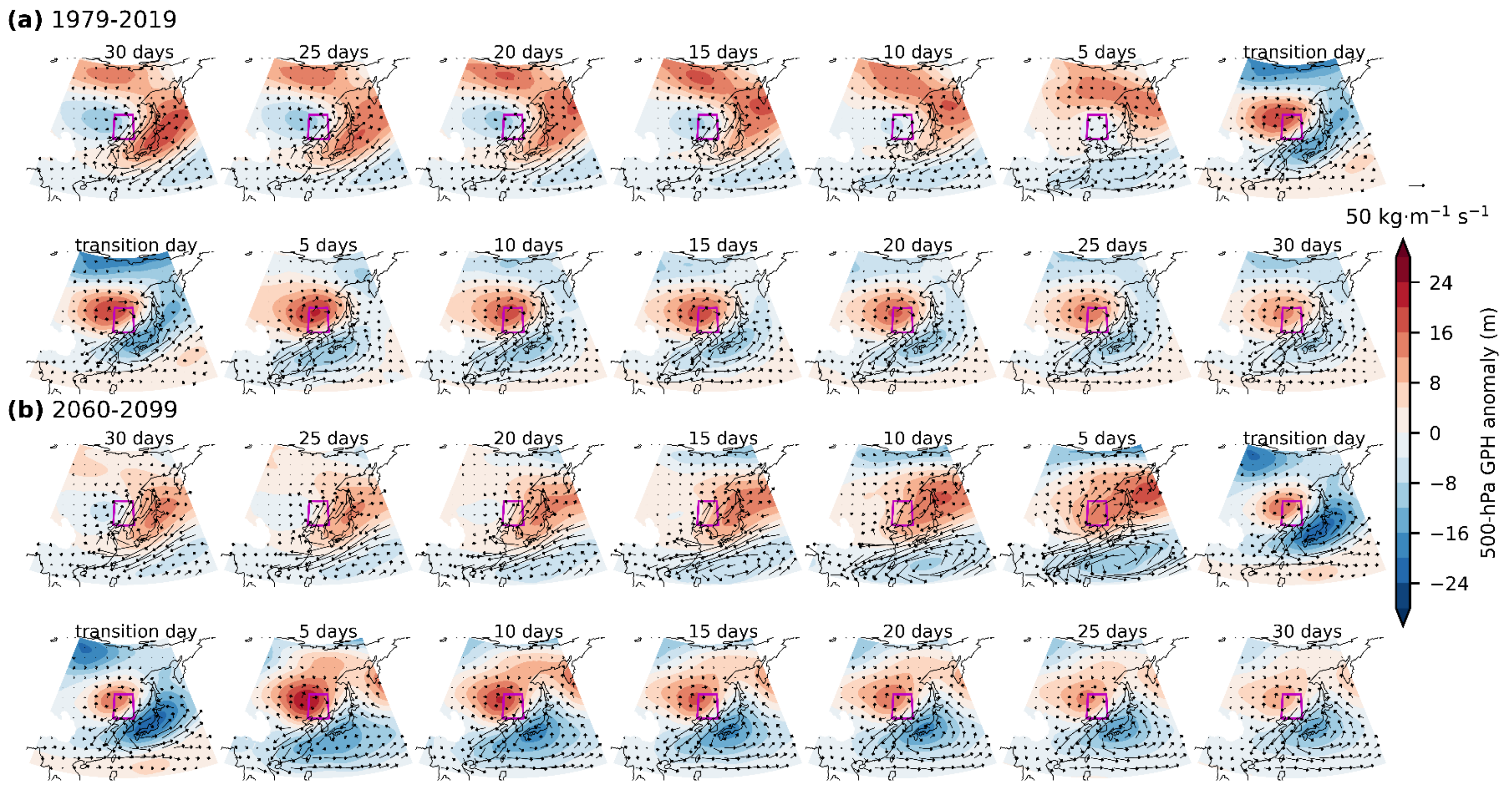

Supplementary Figure 22 Gradual changing process of large-scale atmospheric circulation of wet-to-dry whiplash over northeastern China (NEC). Average atmospheric anomalies for the 30, 25, 20, 15, 10 and 5 days before the occurrence of whiplash, the transition days, and the 5,10, 15, 20, 25, 30 days after the occurrence over the current period (1979-2019; **a**) and future period (2060-2099; **b**) in the CESM-LENS ensemble. Composite 500 hPa geopotential height (GPH) anomalies are shown by color and contour lines, and the magnitude and direction of the column-integrated water vapor anomalies are shown by scaled arrows.

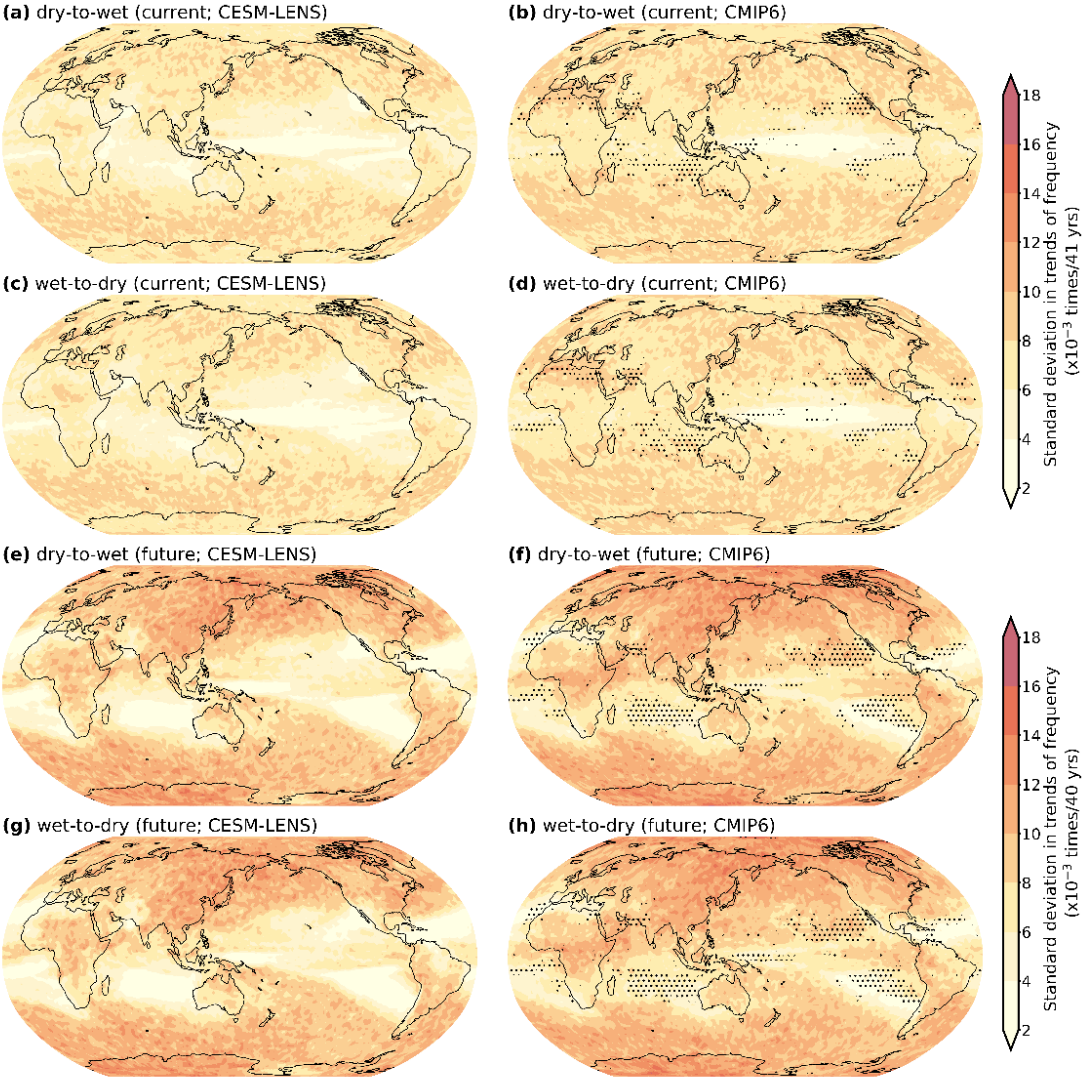

Supplementary Figure 23 Global maps of standard deviation in frequency trends of whiplash for the (top two rows) current period (1979-2019) and (bottom two rows) future period (2060-2099). Trends are shown for both the 40-member CESM-LENS ensemble and the 54-member CMIP6 ensemble. Stippling on the CMIP6 maps indicates standard deviations that are statistically different than the CESM-LENS for the corresponding period. Stippling is based on the *f* test and a 95% confidence interval.

Supplementary **Table 1**. Information of 22 CMIP6 models used in this study.

Model	Institution	Lat × Lon	The Number of
ACCESS-ESM1-5	CSIRO	192×145	3
CanESM5	CCCma	128×64	6
CESM2-WACCM	NCAR	288×192	5
CMCC-CM2-SR5	CMCC	288×192	1
CMCC-ESM2	CMCC	288×192	1
EC-Earth3	EC-Earth-Consortium	512×256	2
EC-Earth3-CC	EC-Earth-Consortium	512×256	1
EC-Earth3-Veg	EC-Earth-Consortium	512×256	4
EC-Earth3-Veg-LR	EC-Earth-Consortium	320×160	3
GFDL-CM4	NOAA-GFDL	288×180	1
GFDL-ESM4	NOAA-GFDL	288×180	1
INM-CM4-8	INM	180×120	1
INM-CM5-0	INM	180×120	1
IPSL-CM6A-LR	IPSL	144×143	4
KIOST-ESM	KIOST	192×96	1
MIROC6	MIROC	256×128	5
MPI-ESM1-2-HR	MPI-M	384×192	2
MPI-ESM1-2-LR	MPI-M	192×96	5
MRI-ESM2-0	MRI	320×160	5
NorESM2-LM	NCC	144×96	1
NorESM2-MM	NCC	288×192	1
TaiESM1	AS-RCEC	288×192	1

Supplementary **Table 2.** Summary of the 6 gridded precipitation datasets used in this study. Abbreviations in the data source(s) column defined as follows: G, gauge; S, satellite; and R, reanalysis.

	Dataset	data source(s)	Spatial resolution	Coverage	Period used
1	ERA-5	R	0.25° × 0.25°	Global	1979-2019
2	MERRA-2	R	0.5°× 0.625°	Global	1980-2019
3	JRA-55	R	1.25° × 1.25°	Global	1979-2019
4	CHIRPS	G, S, R	0.05° × 0.05°	50° S-50° N Land only	1981-2019
5	GPCC	G	1° × 1°	Land only	1982-2019
6	REGEN_LongTermStns	G	1° × 1°	Land only	1979-2016

Supplementary References

1 Deser, C., Phillips, A., Bourdette, V. & Teng, H. Uncertainty in climate change projections: the role of internal variability. *Climate Dynamics* **38**, 527-546, doi:10.1007/s00382-010-0977-x (2012).

2 Tebaldi, C., Arblaster, J. M. & Knutti, R. Mapping model agreement on future climate projections. *Geophys. Res. Lett.* **38**, L23701 (2011).

3 Kay, J. E. *et al.* The Community Earth System Model (CESM) large ensemble project: A community resource for studying climate change in the presence of internal climate variability. *Bulletin of the American Meteorological Society* **96**, 1333-1349 (2015).
